# Salivary MicroRNAs as Innovative Biomarkers for Diagnosis and Prediction of the Oral Potentially Malignant Disorders Transition Towards Oral Cancer: A Systematic Review

**DOI:** 10.3390/jcm14228128

**Published:** 2025-11-17

**Authors:** Ciprian Osan, Ioana Berindan-Neagoe, Cristian Dinu, Gabriel Armencea, Simion Bran, Winfried Kretschmer, Grigore Baciut, Florin Onisor, Mihaela Baciut

**Affiliations:** 1 Department of Maxillofacial Surgery and Implantology, “Iuliu Hatieganu” University of Medicine and Pharmacy, 400033 Cluj-Napoca, Romania; cipri.osan@yahoo.com (C.O.);; 2 Research Center for Functional Genomics, Biomedicine and Translational Medicine, “Iuliu Hatieganu” University of Medicine and Pharmacy, 400033 Cluj-Napoca, Romania; 3 Klinik fur Mund-, Kiefer- und Plastische Gesichtschirurgie, Alb Fils Kliniken GmbH, 73035 Goppingen, Germany

**Keywords:** oral potentially malignant disorders, oral cancer, salivary microRNA, biomarker, saliva

## Abstract

**Background/Objectives**: Oral potentially malignant diseases (OPMD) are a group of specific conditions characterized by a variable degree of progression to oral cancer. Although these lesions are generally easily recognizable, clinicians face a difficult challenge in predicting which lesions will undergo malignancy. This fact becomes more pressing when considering that early detection of OPMD significantly influences the survival toll. Our systematic review aims to evaluate current evidence of the mechanism through which salivary microRNAs are involved in OPMD and to study the possibility of using these molecules as a novel biomarker for predicting transition to oral cancer. **Methods**: A comprehensive search in PubMed, Google Academic, Cochrane and Scopus databases was performed, analyzing studies conducted between 2014 and 2025. The quality of studies was evaluated using Quality Assessment of Diagnostic Accuracy Studies (QUADAS-2). **Results**: A total of 1046 articles were found; 76 articles were thoroughly examined, but only 33 articles were included in this systematic review. Salivary microRNAs, such as miR-21, miR-34a or miR-320a, were found to be dysregulated in OPMD samples compared to healthy and oral squamous cell carcinoma samples (OSCC), contributing to malignancy through gene expression alteration. **Conclusions**: Salivary microRNAs were found to be intricately involved in the malignant transformation of OPMD, potentially being promising biomarkers for early detection of oral cancer.

## 1. Introduction

Despite the progress made in the field of oncology over the last decades, cancer remains a major worldwide health dilemma, with approximately 10 million deaths occurring in 2020 [1]. Being an utterly heterogeneous disease, cancer still encourages ardent controversies in the scientific field regarding the most effective method of inhibiting the anarchic proliferation of cells. Conventional treatments, involving surgery, chemo/radiotherapy and immunotherapy, continue to be the main approaches for treatment, but tumor relapse and adverse effects of the therapies still pose a challenge [2].

As a definition, oral potentially malignant disorders (OPMD) are a group of mucosal disorders with variable degree of progression to oral cancer [3]. These lesions represent an intermediate stage in the progression towards oral cancer, indicating that they have only suffered a small amount of the genetic changes observed in OSCC [4]. A wide range of pathologies such as leukoplakia, erythroplakia, oral lichen planus and submucous fibrosis can be included in the category of premalignant lesions. Even though the majority of these disorders regress or remain stationary, a variable number of OPMD develop genetic alteration and microenvironment changes, which, ultimately, conduct to malignant transformation [5,6]. For instance, leukoplakia carries an associated risk of 3.3% for cancer development over 5 years [7]. Tissue biopsy remains the golden standard for differentiation between premalignant lesions and early OSCC, because it can not only distinguish the histopathological type of neoplasms, but it can also reveal its degree of differentiation [8]. However, in some cases, it becomes difficult to procure biopsy material due to localization of certain tumors, and in eventuality of multifocal lesions, several tissue samples are required. Therefore, there is an acute need to develop novel biomarkers capable of non-invasive identification of malignancy OPMD in order to improve the outcome of patients.

Oral cancer is described as one of the most common malignancies in developing countries, with an elevated incidence in Melanesia, South Central Asia and Eastern Europe [9]. In 2020, nearly 177,757 deaths were recorded with oral cancer as the primary cause [1]. This precarious prognosis, with an overall 5-year survival rate that varies between 40 and 80%, is caused by tardive diagnosis and the frequent tumor recurrence [10]. The most predominant subtype is squamous cell carcinoma (OSCC), with more than 90% of all oral cancers having this origin [11]. Tobacco smoking, alcohol consumption and HPV infection are frequent risk factors that are strongly associated with oral carcinogenesis, disturbing local homeostasis by inducing DNA mutation and genetic alteration [12].

MicroRNAs are short (22–23 nucleotides), non-coding RNAs, capable of regulating gene expression, with an incremental involvement in cancer pathogenesis [13]. There are two main mechanisms by which microRNAs interfere post-transcriptionally with their RNA targets. The first one involves the imperfect complementary binding within the 3′-untranslated regions (3′-UTRs) of target mRNAs, while the second one includes perfect complementarity binding to mRNA sequences, leading the degradation of target mRNAs [14]. By modulating the levels of genes, microRNAs play an important role in cell growth, differentiation, apoptosis and interactions with local environment [15,16]. Circulating microRNAs can be defined as the miRNAs that are secreted extracellularly in various biofluids including serum, plasma, saliva, breast milk, ascites fluid and others [17]. Following transcription and maturation of these molecules, microRNAs are expelled into extracellular medium in 4 possible forms: within the exosomes by active exocytosis, through the export of apoptotic bodies [18], as shedding vesicles [19] or in association with RNA-binding proteins (AGO 1–4) [20] or high-density lipoproteins [21]. Once they enter the external microenvironment, microRNAs continue to be involved in the hallmarks of cancer, regulating proliferation, differentiation, apoptosis, angiogenesis, local invasion and metastasis formation [22].

The purpose of the current systematic review is to evaluate the possibility of using the expression level of salivary microRNAs as a tool for early detection of OPMD in order to prevent all the complications associated with the development of OSCC. In addition, this scientific paper aims to present the most relevant mechanism involved in OPMD transition towards OSCC, along with the major advantages of using microRNAs for detecting malignancy of those lesions.

## 2. Materials and Methods

The PRISMA checklist (Supplementary Materials) was utilized as a guideline to organize and design this systematic review [23,24].

### 2.1. Eligibility Criteria

This research paper included all studies that evaluated the difference in salivary microRNAs level of expression between healthy individuals, patients with premalignant lesions and patients with OSCC. Also, PICOS terms were used in order to conduct a thorough investigation: participants (patients with malignant or premalignant lesions), intervention (dosage of altered level of salivary microRNAs in saliva of oral potentially malignant disorders or OSCC patients), control (microRNAs expression in healthy subjects) and outcome (difference in salivary microRNAs level of expression between healthy subjects, patients with premalignant lesions and OSCC ones), and the study design included cross-sectional studies, prospective comparative studies, retrospective cohort studies and case–control studies.

Inclusion criteria were as follows:

(1) Studies that analyzed the salivary microRNAs expression in OPMD and oral cancer patients;
(2) Text available in full format;
(3) Articles written in English;
(4) Respecting the structure of IMRAD (introduction, material and method, results, discussions);

Exclusion criteria were as follows:

(1) Reviews, meta-analysis, case reports, case series, opinion articles and letters to the editor;
(2) Articles that incorporate various forms of treatment administration for OPMD a priori to saliva sample acquisition;
(3) Studies that include patients with other oral cavity tumors (non-OSCC) or in other regions of the organism, patients with history or undergoing chemo/radiotherapy and patients with immunosuppression;
(4) Duplicated publications;
(5) Animal experiments;

The process used to determine the eligibility of each study for the planned syntheses involved analyzing study characteristics, including the specific microRNA(s) investigated, detection methods, type of lesion (OPMD or OSCC), presence of a healthy control group, and the reported expression levels. Based on these characteristics, studies were allocated to one or more of the three comparative syntheses: healthy vs OPMD, healthy vs OSCC, and OPMD vs OSCC. A study was included specific synthesis only if it reported microRNA expression levels for both groups required for that specific comparison.

### 2.2. Publication Search Strategy

An electronic search on Pubmed, Google Academic, Cochrane and Scopus databases was performed. Appropriate keywords and Medical Subject Headings (MeSH) terms were selected and systematically combined using Boolean operators, such as “AND”, “OR”.

Published papers on level of expression of salivary microRNAs in premalignant lesions from 2014 to 2025 were found using the following combination of keywords: (salivary microRNA OR exosome microRNA OR circulating microRNA OR extracellular microRNA) AND (oral cancer OR oral squamous cell carcinoma OR oral premalignant lesions OR leukoplakia OR oral lichen planus OR erythroplakia OR oral submucous fibrosis OR oral potentially malignant disorder). Two independent reviewers (C.O., G.A.) extracted data available on study characteristics (article characteristics, sample types, saliva collection protocol, microRNA extraction and analysis, name of microRNA(s) and type of dysregulation, main study results like sensitivity, specificity and conclusion). All of the inconsistencies were further discussed, and a consensus was obtained. The extracted data focused on identifying changes in microRNA expression levels in both OSCC and OPMD and examining their correlation with the transition from a non-malignant lesion to oral cancer.

### 2.3. Qualitative Evaluation

In order to minimize the risk of bias, all of the included research papers were subjected to a qualitative assessment using Quality Assessment of Diagnostic Accuracy Studies (QUADAS-2). This tool consists of four key domains: Patients Selection, Index test(s), Reference Standard and Flow and Timing. Every article included was thoroughly analyzed by the two independent examinators (C.O., G.A.), and every domain was rated either “high”, “unclear” or “low” according to their bias risk. QUADAS-2 tool provides a series of “signaling” questions which guide the researchers in proper evaluation of bias risk. If all of the signaling questions in a domain are answered “yes,” the risk of bias is judged as low. Conversely, if signaling questions are answered “no”, the domain is considered to have a potential risk of bias. In addition, using QUADAS-2 tool, the examiners performed an applicability assessment of the reviewed articles [25]. Further details regarding the steps involved in qualitative evaluation of studies can be found in Table 1.

## 3. Results

### 3.1. Literature Search and Study Selection

**Figure 1:**
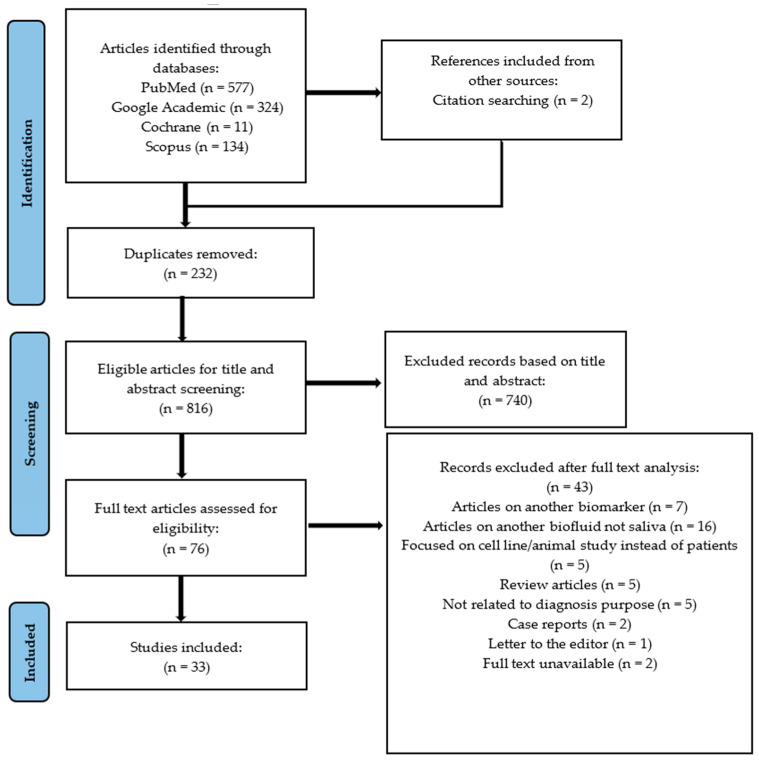
PRISMA flow-chart diagram for the selection of 33 papers included in the review.

After the initial search, a total of 1046 scientific articles were retrieved from databases (577 from Pubmed, 324 from Google Academic, 11 from Cochrane database, 134 from Scopus). The duplicates were removed (232), and the other articles were analyzed by two independent authors (C.O., G.A.) according to the inclusion and exclusion criteria. A total of 76 articles were declared potentially admissible and underwent an integral text examination, but only 33 articles entirely met our criteria and were included in this systematic review. Further details regarding the main reasons for study exclusions can be found in Figure 1.

### 3.2. Characteristics of the Included Studies

The design of the studies was slightly different: 10 studies focused on highlighting the distinct salivary microRNA expression between premalignant lesions and healthy subjects, 9 studies focused on salivary microRNA levels between OSCC and healthy individuals, whereas 14 articles compared the levels of microRNA from the saliva of patients with premalignant lesions to patients with OSCC, in conjunction with saliva sampled from healthy individuals. A detailed presentation of the included studies is provided in Table 2. Moreover, no statistical methods such as subgroup analysis or meta-regression were used to explore heterogeneity, as a meta-analysis was not feasible. Potential sources of heterogeneity were instead examined qualitatively by comparing differences in study design, sample collection protocols, microRNA detection techniques and reporting formats across studies.

### 3.3. Quality Assessment

The articles underwent a qualitative assessment through QUADAS-2 tool analysis. Following the analysis, a significant number of articles raised concerns related to Patient Selection domain (Table 3). The most frequent issues encountered in the patient selection domain were the small sample size (usually less than 50 patients) [26,27,29,33,34,35,36,43,45,51,55,57], which could impact the statistical stability and the confidence intervals. Another issue observed was the demographic imbalance, as the OPMD and OSCC population were significantly older than the controls and predominantly male. The main concerns regarding the index test were the selective reporting (only miRNA with significant results were presented), the lack of blinding and post hoc derivation of diagnostic cut-offs. The reference standard and flow and timing domains did not, for the most part, induce a significant risk of bias.

### 3.4. Saliva Characteristics and Investigative Techniques

Saliva is considered to be one of the most valuable, non-invasive biofluids of the human body. Being loaded with immunoglobulins, enzymes, mucins, electrolytes and genetic material, saliva fulfills multiple roles starting from being a truly antiviral and antimicrobial agent and ending with being a protective assistant against erosion and demineralization of teeth [59]. Nowadays, more and more scientists focus on a more inconspicuous role of this biofluid, namely on the possibility of using it as a biomarker for non-invasive cancer detection and monitoring [60]. For instance, the presence of increased levels of IL-10 and IL-13 can be an indicative marker for existence of OSCC [61]. The same outcome can be visualized when comparing the expression of salivary miR-365 from an OSCC sample to a control one. Not only an elevated level of salivary miR-365 was found in exosomes from OSCC compared with normal oral cell lining, but it could also be correlated with disease stage [62,63]. Also, it is important to highlight the advantages of using saliva as a liquid biopsy: low-cost, rapid method of sample collection, non-invasive character, does not require special personnel and storage and is less sensitive than other sample types [64].

In scientific papers included in this systematic review, all the experiments were conducted using whole saliva (Table 4). Moreover, 21 studies specified the use of unstimulated saliva detrimental to stimulated saliva [26,28,29,30,32,34,35,37,41,43,44,45,48,49,50,51,52,53,54,55,58]. A possible explanation is that unstimulated saliva is often taken as the average representative of the entire ecosystem of the oral cavity, represented by all three major salivary glands, minor salivary glands as well as gingival crevicular fluid secretions, and that it does not require any previous preparation [65]. In contrast, two studies focused on salivary microRNA identification from oral swirl [38,39]. Requesting the patients to swirl sterile deionized water or PBS for 60 s allows a more uniformized sampling method and significantly reduces the time required for saliva sampling [66]. Moreover, one study used as a primary method for salivary microRNA sampling oral swabbing [42].

Information regarding accurate instructions provided to patients before saliva collection was extracted from included studies. More specifically, in 19 of the included articles, patients were informed to refrain from consuming food, drinking or performing any oral hygiene procedures for 30 min to 2 h prior to sampling [26,28,31,32,33,35,37,40,41,43,44,48,50,51,52,53,56,57,58]. In addition, in 6 studies patients were asked to abstain from tobacco use for times ranging from 30 min to 1 day before the saliva sampling [40,41,44,48,50,58]. In order to preserve the integrity of RNAs, RNA Protect Saliva Reagent products were used in 9 studies [27,32,37,41,48,50,53,54,57]. Regarding the storage of saliva, the most common temporary storage was at room temperature, the samples being processed in the shortest time [26,30,31,40,43,44,48,49], while the most frequent long-term storage was at −80 °C [26,27,30,31,32,33,34,35,37,43,44,45,46,47,48,51,52,53,54,56,57,58]. Another aspect worth mentioning is the quantity of saliva used for these experiments. Moataz M ELHefny and his team were able to obtain the expression level of salivary microRNAs in different types of samples using only 0,4 mL of saliva, proving that a limited amount of saliva samples contains satisfactory quantity of microRNAs for analysis [58]. In other experiments, an equally reduced volume of saliva was employed between 0.5 and 10 mL [44,48,50,52,53,54,57].

### 3.5. Salivary MicroRNAs Expression in OPMD vs. Healthy Controls

In the articles included in this systematic review, salivary miRNAs expression were found to be dysregulated in samples analyzed from OPMD patients compared to healthy controls. In more exact terms, salivary miR-21, miR-31, miR-93, miR-142-3p, miR-146a, miR-155, miR-181b, miR-184, miR-196b, miR-375, miR-412-3p and miR-4484 were found to be upregulated, while miR-27b, miR-125a, miR-137, miR-138, miR-320a, miR-424 and miR-3928 were downregulated in the saliva of patients diagnosed with premalignant lesions compared to healthy individuals.

Regarding the studies included in this systematic review, it has been noted that 5 articles discovered the expression level of salivary miR-21 to be significantly elevated in OPMD samples compared to healthy individuals. Uma Maheswari found that the level of miR-21 was 2.44 times increased in leukoplakia and 2.03 times in OLP in comparison to saliva taken from control subjects [40]. Concerning the sensitivity and specificity of the tests used to identify OPMD, regarding miR-21, Uma Maheswari’s study observed 66% sensitivity and 69% specificity, which may serve as a reference point for predicting the presence of OPMD (Table 5) [40]. Similar results were obtained by F. Zahran and his collaborators, who noticed that miR-21 was significantly upregulated in both OPMD with or without dysplasia compared to healthy controls [28]. The sensitivity and specificity obtained were more promising. MiR-21 was able to identify the OPMD with an accuracy of 90%, whereas the sensitivity was only 60%. A relatively low sensitivity could be attributed to population or technical variability related to saliva collection and normalization procedures. These results align with recent studies on this topic. For instance, Masoumeh Mehdipour identified that miR-21 levels were significantly increased in saliva samples derived from patients with OLP compared to those from healthy controls, being a genuine biomarker panel to recognize OLP lesions [37]. In addition, Shesha R. Prasad demonstrated that miR-21 could also distinguish a patient suffering from OSMF from healthy individuals [41]. On the contrary, the study presented by Dario Di Stasio did not demonstrate any variation in salivary miR-21 between the sample groups [49].

Even though the results for salivary miR-31 were not statistically significant, an upregulation was observed, with a fold increase of 1.6 times and 1.2 times, respectively, for leukoplakia and OLP when compared to controls [40]. Better results were obtained by Masoumeh Mehdipour. He found that the salivary miR-31 levels were higher in dysplastic OLP patients in comparison to the healthy control group (*p* = 0.01) [37]. Furthermore, Saba Khan reported in his study that miR-31 had significantly higher median fold change of expression level, not only in OLP and leukoplakia as described before, but also in OSMF patients [46]. Regarding the detection of OLP, another two salivary microRNAs could serve as discriminating markers between OLP subjects and healthy ones. For instance, salivary miR-4484 was substantially upregulated (*p*-value = 0.03545) in OLP patients in a range of 2- to 98-fold out of 14 tested patient samples [29]. Equally, overexpression of salivary miR-142-3p could become an encouraging diagnostic biomarker for OLP, as it was detected overexpressed in the saliva and tissue samples collected from patients with OLP [47]. The salivary concentration of miR-181b was different among the dysplastic and non-dysplastic OPMD compared to controls. An upregulation of the above-mentioned microRNA was found in pathological samples, with 5.14-fold increase in dysplasia lesions and 6.29-fold increase for non-dysplastic lesions [49]. Moreover, salivary miR-184 was also up-regulated in OPMD with or without dysplasia when compared to healthy saliva samples [28].

As for downregulated salivary microRNAs, a considerable decrease in miR-34a expression was observed in leukoplakia patients compared to control [36]. Similar results were obtained by Minoo Shahidi: salivary miR-320a was strongly downregulated in OLP with dysplasia probes compared to healthy controls [32]. The same pattern was followed by salivary miR-27b. An under-expression by 4.52-fold of salivary miR-27b was reported in dysplastic OED compared to healthy saliva samples [49]. The same outcome was achieved by Sana Aghbari but in OLP. Salivary miR-27 expression exhibited a statistically significant downregulation in the OLP group, with the mean value of 5.343 (±4.875), while the control group reached 10.592 (±2.142) [34]. The same author discovered that besides miR-27, miR-137 was also under-expressed in patients with OLP [34]. Moreover, Masoumeh Mehdipour found another microRNA that was dysregulated in OLP. More specifically, miR-125a saliva levels were decreased in patients with OLP (*p* <0.001). This result is in accordance with the data obtained by research articles focusing on miR-125a levels in oral cancer [67,68]. Furthermore, salivary levels of miR-145 and miR-375 were significantly decreased in cases of OPMD with or without dysplasia [28,48].

With regard to the sensitivity and specificity of the tests used to identify OPMD, regarding miR-21, Uma Maheswari’s study observed 66% sensitivity and 69% specificity, which may be a base point for predicting local unrest representative to OSCC (Table 5) [40]. For miR-31, poorer accuracy was found, with only 36% sensitivity and 40% specificity [40]. More balanced results were obtained by Dario di Stasio, where the values of sensitivity and specificity of using miR-181b as a biomarker were 94.1% and 81.2%, respectively [49]. Another remarkable data finding was achieved by F. Zahran, where miR-21 was able to identify the OPMD with an accuracy of 90%, whereas the sensitivity was only 60% [28]. On the other hand, miR-145 showed a cutoff point of a 0.6 decrease rate, with 70% specificity and 60% sensitivity, while the cutoff point of miR-184 showed a 3-fold increase, with 75% specificity and 80% sensitivity [28]. Another microRNA with similar characteristics but with inferior precision of recognizing OPMD is miR-375. With 80% sensitivity and 68% specificity, miR-375 could potentially be exploited to detect insidious OPMD in susceptible populations (34). In terms of specific identification of OLP, samples collected from patients suffering from this condition could be diagnosed by using salivary miR-27b, miR-137, and miR-142-3p, with sensitivity ranging from 75% to 100%, and specificity from 80% to 100% [34,47].

These results are encouraging, because they can create the perfect premise for early detection of the disease before the development to OSCC. Even though the potential risk of progression of OPMD to OSCC is relatively reduced, as about 1.36% per year for leukoplakia [69] or 2.7% per year for erythroplakia [70], the risk continues to exist and can lead to insidious development of OSCC. The issue is all the more pressing as tardive detection of OSCC is associated with regional and distant metastasis which diminish the survival rate by 50% [71,72]. Therefore, salivary microRNAs could be used as novel, non-invasive biomarkers capable of detecting OPMD from any part of the oral cavity.

## 4. Discussions

### 4.1. Salivary MicroRNAs as Novel Biomarkers for Diagnosis of OPMD and OSCC

Premalignant lesions are in situ modifications of the normal tissue, with a variable tendency to develop into malignant tumors. These lesions can have multiple localizations across the living organism, with vast clinical and histopathological expression [73,74,75]. In the oral cavity, the most commonly described potentially malignant disorders are leukoplakia, erythroplakia, oral submucous fibrosis and oral lichen planus [76]. The most important aspect that endorses the possibility of using microRNAs as biomarkers is the fact that they exhibit the traits of the tissues from which they originate. This fascinating feature is advantageous in case of insidious onset of premalignant lesions. Even though, in most cases, oral potentially malignant disorders are easily diagnosed, there are several regions where these cannot be easily accessed or visualized, being masked by the anatomical elements of the oral cavity [77]. Early diagnosis followed by a proper treatment is crucial, since it may affect the prognosis of the patient [78]. Therefore, salivary microRNAs could be useful in this particular situation.

Several scientific articles addressed the potential role of using salivary microRNAs as innovative biomarkers for cancer detection and prognosis evaluations [79,80,81]. For instance, salivary miR-31 was significantly upregulated in OSCC patients compared to healthy controls, denoting that the usage of that microRNA could discriminate cancerous lesions from normal tissue. In addition, after tumor resection, both plasma and saliva levels of miR-31 were reduced, indicating that an oral tumor was the main origin for altered microRNA production [82]. Another aspect worth mentioning is that salivary microRNAs can be used as a discriminatory tool for assessing the possibility of performing surgical intervention in pancreatic cancer. Hsa-miR-21 could distinguish the patients with unresectable pancreatic cancer from those with pancreatitis, intraductal papillary mucinous neoplasm or cancer-free patients [80]. Moreover, salivary microRNAs such as exosomal miRNA-1307-5p could be used as an indicator for predicting poor survival and impaired patient outcome in oral cancers and can contribute in clinical staging of the salivary gland neoplasms [51,79].

### 4.2. Salivary MicroRNAs Pathogenesis in Oral Premalignant Lesions Causing Malignant Transformation of OPMD

Histopathological evaluation remains the gold standard for assessing oral potentially malignant lesions. However, the current classification of OPMD with distinct grades of dysplasia cannot precisely predict which of the lesions will suffer a malignant transformation [11]. Thus, salivary microRNAs could be used as an identification method of a subtle turnaround of premalignant lesions towards OSCC.

One of the most suitable examples that depicts the transition between premalignant and malignant states is the expression level of salivary miR-21, quantified in various grades of OPMD dysplasia. Even though in Uma Maeheswari’s study, no direct, linear correlation between OPMD progression from mild or moderate to severe dysplasia could be established, considerable overexpression of salivary miR-21 was observed in OPMD with severe dysplasia [40]. Dysplastic characteristics of oral squamous epithelium gained by OPMD progression are characterized by cellular atypia and abnormal architecture of the epithelium [83]. Clinical changes are also reflected at the molecular and genomic levels, where modification in keratinocytes proliferation and differentiation is driven by genetic alterations by gene alteration and cell cycle perturbance [84]. Another scientific paper included in this systematic review found the same results. A gradual increase in salivary miR-21 expression was observed from healthy mucosa to OPMD without dysplasia, OPMD with dysplasia and OSCC. However, it is worth noting that the values of salivary miR-21 were significantly increased in OPMD with dysplasia and OSCC compared to other groups, delimiting inflammatory lesions from non-dysplastic ones [28]. Dysregulation of miR-21 is recognized as a notable stage in inflammatory response alteration in many types of cancer [85,86,87]. MiR-21 upregulation in colon cancer can be taken as an example. A positive correlation between miR-21 and Interleukin-6 (IL-6) expression was discovered in colon cancer; miR-21 levels could be enhanced by oncogenic signaling IL-6, leading to NF-kB activation, which maintain an inflammatory and a pro-carcinogenic state [88].

Salivary expression level of miR-320a is another example of a gradual transition from OLP to OSCC. A decreased salivary level of miR-320a was found in dysplastic OLP and OSCC probes compared to non-dysplastic OLP and healthy individuals. MiR-320a could potentiate the oral lichen planus progression towards OSCC by modulating the expression of VEGFR-2 [32]. Described as an angiogenic biomarker, VEGFR-2 not only promotes endothelial proliferation, migration and differentiation, but it also increases the proliferative capacities of cancerous cells [89,90]. Therefore, an increase expression of VEGFR-2 creates a favorable environment for tumor development. Moreover, the expression of miR-320a could be strongly associated with elevated levels of salivary IL-6 and salivary C-reactive protein (CRP). Therefore, the more oncogenic changes that occur in oral tissue, the lower levels of salivary miR-320a, and the higher levels of salivary IL-6 and CRP, will be discovered [32]. IL-6 is a component of the tumor microenvironment (TME), which represents all the non-cancerous host cells in the tumor, such as fibroblasts, endothelial cells and immune cells, along with non-cellular components, including extracellular matrix (ECM), cytokines and growth factors [91]. It is important to mention that multiple cell types in TME do not exist in isolation; instead, they form a complex interaction that contributes to tumor initiation and progression of cancer. It was discovered that in chronic inflammation, normal fibroblasts start a conversion process to cancer-associated fibroblasts (CAF) [92]. Having a new secretory profile, CAF produce elevated levels of IL-6 and other pro-tumorigenic cytokines and interact with tumor cells, which in turn secrete high levels of IL-6. These actions result in STAT3 and MEK/ERK signaling pathways, which stimulate tumor cell proliferation, invasion, growth and decrease apoptosis [93]. Taking into consideration that high levels CRP were found to be strongly associated with cancer severity, salivary IL-6 and CRP, along with miR-320a, could be used as indicators for healthy mucosa transition to OLP and, further, to OSCC [94]. Hence, salivary microRNAs are responsible for the microenvironment modification, which creates the premises of cancer onset and development.

Salivary miR-34a, one of the most studied microRNAs involved in the pathogenesis of malignant tumors, was found to be dysregulated in oral leukoplakia. Therefore, downregulation results in elevated expression of CD44v6 and reduced SYNE1, both in patients with leukoplakia and OSCC [36]. CD44v6 is an isoform of CD44, a transmembrane glycoprotein, that possesses the ability to bind hyaluronan, extracellular matrix proteins and growth factors [95]. Bearing in mind that this molecule increases the accumulation of nuclear β-catenin, and thus, enhances cell proliferation and EMT in cancer, it is possible for CD44v6 to be a subtle element of transition between leukoplakia and OSCC [96]. Another microRNA involved in EMT regulation is miR-203, a significantly upregulated microRNA in oral submucous fibrosis compared to normal oral mucosa, which can become a decisive factor for malignant transformation of oral submucous fibrosis by targeting SFRP4 and TM4SF1 [97]. Moreover, in OLP, upregulated miR-155, targets the miR-155/SOCS1 axis, a pathway with high involvement in immune system and in macrophage differentiation, functioning overall as a tumor-promoting factor [98,99].

MiR-181b, overexpressed in patients with OPMD compared to healthy subjects, follows the trend established by the other microRNAs, increasing its salivary level with the increasing degree of dysplasia, supporting its potential as a promising biomarker for tumor risk stratification [49]. On the other hand, miR-181b decreases considerably in OSCC. The difference could be interpreted as a total loss of tissue architecture that occurs in OSCC, which creates the basis for a negative feedback mechanism. In addition, Zhi-Yuan Deng and his team discovered that the expression level of miR-181b was significantly lower in tumor-adjacent tissue compared to oral verrucous carcinoma and normal mucosa. These results suggest that miR-181b expression could be associated with the degree of malignancy, showing that oral verrucous carcinoma is less predisposed to invasion and metastasis than OSCC [100]. These apparently contradictory results, with miR-181b expression increasing with the degree of dysplasia and downregulated in OSCC and oral verrucous carcinoma, from the past two studies presented could also be explained by the distinct study designs (in vivo or ex vivo) and contrasting sampling targets (saliva vs. tissue) [49].

Being a noninfectious, chronic inflammatory disorder, OLP is described as having aberrant miRNA expression levels [101,102]. For instance, the expression of miR-137 was markedly downregulated in the oral mucosa of OLP patients and showed an inverse correlation with CD8 tissue expression, indicating immunomodulatory functions of miR-137 [103]. In a study conducted by Hsi-Feng Tu, luciferase reporter assay results indicate that salivary miR-142-3p could target glucocorticoid receptor α (GRα) gene [48]. GRα is a member of the steroid receptor family, which regulates the action of glucocorticoids [104]. As the predominant isoform, GRα is involved in a wide range of pathological processes, including inflammation, immune response and cellular differentiation [105]. The GRα downregulation produced by miR-142-3p action is a pertinent marker for the disturbance of anti-inflammatory function of the cells, which may support the malignancy of OLP [106].

In another study related to this topic, 45 patients with leukoplakia were treated with 13-*cis*-retinoic acid and were monitored for approximately 3 years. During this period, 10 patients developed carcinoma in situ or OSCC with an average time of 30.1 months and the other 7 remained at the leukoplakia stage. The other patients were excluded for various reasons [107]. Remarkably is that 25 microRNAs were differentially expressed between progressive and non-progressive leukoplakias.

The included evidence has several limitations, most notably small sample sizes, substantial heterogeneity in saliva collection and microRNA detection methods, and inconsistent or incomplete reporting of quantitative data. Many studies selectively reported only significant microRNAs and lacked precision measures such as confidence intervals, increasing the potential for reporting bias. Demographic imbalances between cases and controls further limit comparability. These issues reduce confidence in the overall evidence base.

## 5. Conclusions

In summary, the discovery of a tailored treatment for patients with oral premalignant changes, according to their molecular profile, is crucial. The pressing challenge is to determine which OPMD will progress to OSCC and which will not. Salivary microRNAs could represent a solid answer to this matter. Expression changes in microRNAs are increasingly recognized as relevant to distinguish the malignant lesions from the premalignant ones without transformation risk. Having the ability to reflect molecular changes in OPMD, even in non-invasive, inexpensive and easily accessible biofluids such as saliva, microRNAs represent a crucial standpoint in screening and detecting OPMD. In this systematic review, several microRNAs such as miR-21, miR-31 and miR-184 could represent promising biomarkers for salivary diagnosis of OPMD malignancy.

## Figures and Tables

**Table 1:** The QUADAS-2 tool signaling questions that assist researchers in systematically assessing the quality and risk of bias in the selected studies.

	Risk of Bias				Applicability Concerns		
Domains	Patient Selection	Index Test	Reference Standard	Flow and Timing	Patient Selection	Index Test	Reference Standard
Signaling questions	Could the Selection of Patients Have Introduced Bias?	Could the Conduct or Interpretation of the Index Test Have Introduced Bias?	Could the Reference Standard, Its Conduct, or Its Interpretation Have Introduced Bias?	Could the Patient Flow Have Introduced Bias?	Are There Concerns That the Included Patients and Setting Do Not Match the Review Question?	Are There Concerns That the Index Test, Its Conduct, or Its Interpretation Differ from the Review Question?	Are There Concerns That the Target Condition as Defined by the Reference Standard Does Not Match the Question?

**Table 2:** General characteristics of the included papers.

No	Title	Author	Year	Number of Participants		Reference
				OPMD or OSCC Group	Control Group	
1	Genomewide Study of Salivary MicroRNAs for Detection of Oral Cancer	F. Momen-Heravi	2014	9 patients with OSCC, 8 patients with OSCC in remission, 8 patients with OLP	9 healthy controls	[26]
2	Difficulties detecting miRNA-203 in human whole saliva by the use of PCR	Martin Lundegard	2015	7 patients with OLP	14 healthy controls	[27]
3	Salivary microRNAs in oral cancer	F. Zahran	2015	20 patients with OPMD with dysplasia 20 patients with OPMD without dysplasia 20 patients with OSCC	20 healthy controls	[28]
4	Diagnostic profiling of salivary exosomal microRNAs in oral lichen planus patients	Jin-Seok Byun	2015	16 patients with OLP	8 healthy controls	[29]
5	MicroRNA-31 upregulation predicts increased risk of progression of oral potentially malignant disorder	Kai-Feng Hung	2016	46 patients OPMD (hyperkeratosis, epithelial hyperplasia or dysplasia)	24 healthy controls	[30]
6	Identification of miR-139-5p as a saliva biomarker for tongue squamous cell carcinoma: a pilot study	Mehmet Bugrahan Duz	2016	25 Tongue squamous cell carcinoma	25 healthy subjects	[31]
7	Predictive value of salivary microRNA-320a, vascular endothelial growth factor receptor 2, CRP and IL-6 in Oral lichen planus progression	Minoo Shahidi	2017	32 patients with OLP, 15 patients with OSCC	15 healthy subjects	[32]
8	Study to explore the significance of saliva as a diagnostic tool to detect microRNA in oral potentially malignant disorders	T.N. Uma Maheswari	2017	5 patients with OPMD (oral leukoplakia, oral lichen planus and oral submucous fibrosis)	5 healthy controls	[33]
9	Evaluating the accuracy of microRNA27b and microRNA137 as biomarkers of activity and potential malignant transformation in oral lichen planus patients	Sana Maher Hasan Aghbari	2018	20 patients with diagnosed OLP	20 healthy individuals	[34]
10	Salivary extracellular vesicle-associated miRNAs as potential biomarkers in oral squamous cell carcinoma	Chiara Gai	2018	21 patients with OSCC	11 healthy controls	[35]
11	Uncovering the potential of CD44v/SYNE1/miR34a axis in salivary fluids of oral cancer patients	Kavan Shah	2018	8 patients with OSCC, 10 patients with leukoplakia	10 healthy subjects	[36]
12	Diagnostic and prognostic relevance of salivary microRNA-21, -125a, -31 and -200a levels in patients with oral lichen planus—a short report	Masoumeh Mehdipour	2018	30 OLP patients 15 OSCC patients	15 healthy subjects	[37]
13	Predicting the Presence of Oral Squamous Cell Carcinoma Using Commonly Dysregulated MicroRNA in Oral Swirls	Tami Yap	2018	30 patients with OSCC	30 healthy subjects	[38]
14	Non-invasive screening of a microRNA-based dysregulation signature in oral cancer and oral potentially malignant disorders	Tami Yap	2019	74 patients with OPMD (OLP, OLL, oral leukoplakia, dysplasia, traumatic ulceration) 53 patients with OSCC	54 healthy subjects	[39]
15	Expression profile of salivary micro RNA-21 and 31 in oral potentially malignant disorders	Uma Maheswari	2020	12 patients with OSMF, 8 patients with leukoplakia, 9 patients with OLP, 7 patients with OSMF + leukoplakia	36 healthy subjects	[40]
16	Expression of Salivary miRNA 21 in Oral Submucous Fibrosis (OSMF): An Observational Study	Shesha R. Prasad	2020	61 patients with chewing habits (chewing gutkha or other forms of areca nut) with OSMF 61 patients with chewing habits without OSMF	63 healthy subjects	[41]
17	Systemic investigation identifying salivary mir-196b as a promising biomarker for early detection of head-neck cancer and oral precancer lesions	Cheng Ann-Joy	2021	30 patients with OPMD 86 patients with HNSCC	52 healthy individuals	[42]
18	Genome-wide study of salivary miRNAs identifies miR-423-5p as promising diagnostic and prognostic biomarker in oral squamous cell carcinoma	Chiara Romani	2021	28 patients with OSCC	14 healthy controls	[43]
19	Salivary miR-30c-5p as Potential Biomarker for Detection of Oral Squamous Cell Carcinoma	Nikolay Mehterov	2021	33 patients with OSCC	12 healthy individuals	[44]
20	Expression of miR-31 in saliva-liquid biopsy in patients with oral squamous cell carcinoma	Parma Kumari	2021	19 patients with OSCC	2 healthy controls	[45]
21	Expression of Salivary miRNA-31 in Oral Submucous Fibrosis	Saba Khan	2021	25 patients with OSMF	25 healthy controls	[46]
22	Clinical significance of miR-142-3p in oral lichen planus and its regulatory role in keratinocyte proliferation	Zhichao Meng	2021	56 OLP patients	44 healthy subjects	[47]
23	Exploiting salivary miR-375 as a clinical biomarker of oral potentially malignant disorder	Hsi-Feng Tu	2022	41 patients with OPMD	26 healthy subjects	[48]
24	Salivary miRNAs Expression in Potentially Malignant Disorders of the Oral Mucosa and Oral Squamous Cell Carcinoma: A Pilot Study on miR-21, miR-27b, and miR-181b	Dario Di Stasio	2022	6 patients with epithelial hyperkeratosis with no dysplasia, 7 patients with low-grade OED 10 patients with high-grade OED 10 patients with OSCC	10 healthy subjects	[49]
25	Salivary miR-31-5p, miR-345-3p, and miR-424-3p Are Reliable Biomarkers in Patients with Oral Squamous Cell Carcinoma	Beáta Scholtz	2022	43 patients with OSCC	44 healthy controls	[50]
26	Salivary Exosomal miRNA-1307-5p Predicts Disease Aggressiveness and Poor Prognosis in Oral Squamous Cell Carcinoma Patients	Aditi Patel	2022	12 patients with OSCC	7 healthy individuals	[51]
27	Salivary Exosomal MicroRNA-486-5p and MicroRNA-10b-5p in Oral and Oropharyngeal Squamous Cell Carcinoma	Cosmin Ioan Faur	2022	25 patients with OSCC	25 healthy individuals	[52]
28	Salivary level of microRNA-146a and microRNA-155 biomarkers in patients with oral lichen planus versus oral squamous cell carcinoma	Masoumeh Mehdipour	2023	15 OLP patients with dysplasia 15 OLP patients without dysplasia 15 OSCC patients	15 healthy controls	[53]
29	Evaluation of Diagnostic Significance of Salivary miRNA-184 and miRNA-21 in Oral Squamous Cell Carcinoma and Oral Potentially Malignant Disorders	Aarushi Garg	2023	30 patients with OPMD 30 patients with OSCC	30 healthy controls	[54]
30	Salivary LINC00657 and miRNA-106a as diagnostic biomarkers for oral squamous cell carcinoma, an observational diagnostic study	Nayroz Abdel Fattah Tarrad	2023	12 patients with OLP 12 patients with OSCC	12 healthy individuals	[55]
31	Downregulation of salivary miR-3928 as a potential biomarker in patients with oral squamous cell carcinoma and oral lichen planus	Alieh Farshbaf	2024	30 patients with OLP 31 patients with OSCC	30 healthy individuals	[56]
32	Expression Analysis of Circulating microRNAs in Saliva and Plasma for the Identification of Clinically Relevant Biomarkers for Oral Squamous Cell Carcinoma and Oral Potentially Malignant Disorders	Federica Rocchetti	2024	6 patients with OPMD 14 patients with OSCC	5 healthy individuals	[57]
33	The oncogenic potential of salivary microRNA-93 and microRNA-412-3p in oral lichen planus: a case–control study	Moataz M ELHefny	2024	20 patients with OLP 20 patients with OSCC	20 healthy controls	[58]

OPMD = Oral potentially malignant disorders; OSCC = Oral Squamous Cell Carcinoma; OLL = Oral lichenoid lesion; OSMF = Oral submucous fibrosis; OLP = Oral lichen planus; OED = Oral epithelial dysplasia.

**Table 3:** Risk assessment of bias and clinical applicability.

Study	Risk of Bias				Applicability Concerns		
	Patient Selection	Index Test	Reference Standard	Flow and Timing	Patient Selection	Index Test	Reference Standard
F. Momen-Heravi 2014 [26]	−	−	+	+	−	+	+
Martin Lundegard 2015 [27]	+	+	+	+	+	+	+
F. Zahran 2015 [28]	+	+	+	+	+	+	+
Jin-Seok Byun 2015 [29]	+	+	+	+	+	+	+
Kai-Feng Hung 2016 [30]	−	−	+	+	+	+	+
Mehmet Bugrahan Duz 2016 [31]	−	−	+	+	−	+	+
Minoo Shahidi 2017 [32]	−	−	+	+	+	+	+
T.N. Uma Maheswari 2017 [33]	−	−	+	?	−	−	+
Sana Maher Hasan Aghbari 2018 [34]	−	−	+	+	+	+	+
Chiara Gai 2018 [35]	−	+	+	+	+	+	+
Kavan Shah 2018 [36]	−	−	+	+	+	+	+
Masoumeh Mehdipour 2018 [37]	+	−	+	+	+	+	+
Tami Yap 2018 [38]	+	+	+	+	+	+	+
Tami Yap 2019 [39]	+	+	+	+	+	+	+
Uma Maheswari 2020 [40]	+	−	+	+	+	+	+
Shesha R. Prasad 2020 [41]	+	+	+	?	+	+	+
Cheng Ann-Joy 2021 [42]	+	+	+	+	+	+	+
Chiara Romani 2021 [43]	−	+	+	+	+	+	+
Nikolay Mehterov 2021 [44]	+	+	+	+	+	+	+
Parma Kumari 2021 [45]	−	−	+	+	−	−	+
Saba Khan 2021 [46]	−	+	+	+	+	+	+
Zhichao Meng 2021 [47]	+	+	+	+	+	+	+
Hsi-Feng Tu 2022 [48]	+	+	+	+	+	+	+
Dario Di Stasio 2022 [49]	+	−	+	+	+	+	+
Beáta Scholtz 2022 [50]	+	+	+	+	+	+	+
Aditi Patel 2022 [51]	−	+	+	+	+	+	+
Cosmin Ioan Faur 2022 [52]	+	+	+	+	+	+	+
Masoumeh Mehdipour 2023 [53]	+	+	+	+	+	+	+
Aarushi Garg 2023 [54]	+	+	+	+	+	+	+
Nayroz Abdel Fattah Tarrad 2023 [55]	−	+	+	+	+	+	+
Alieh Farshbaf 2024 [56]	+	+	+	+	+	+	+
Federica Rocchetti 2024 [57]	−	+	+	+	+	+	+
Moataz M ELHefny 2024 [58]	+	+	+	+	+	+	+

+ Low risk; − High Risk; ? Unclear.

**Table 4:** Saliva collection and microRNA extraction.

No	Author	Sample Areas	Type of Saliva	Quantity of Saliva (mL)	Aspects of Collections	RNA Extraction	RNA Method of Analysis
1	F. Momen-Heravi 2014 [26]	Saliva	Whole unstimulated saliva	8	Condition: refrain from eating, drinking and oral hygiene procedures on the day of saliva collection; saliva was collected from 6 to 11 am, after a water rinse Preservation: - Temporary storage: all saliva samples were processed immediately Long-term storage: −80 °C	miRNeasy kit (Qiagen, Valencia, CA, USA)	NanoDrop Spectrophotometer ND2000 (NanoDrop Technologies, Wilmington, DE, USA), Agilent 2100 Bioanalyzer (Agilent Technologies, Santa Clara, CA, USA), NanoString nCounter miRNA expression assay (NanoString Technologies Seattle, WA, USA), TaqMan MicroRNA Assay (Applied Biosystems) using qRT-PCR (MJ Research PTC-200 Thermal Cycler, MJ Research)
2	Jin-Seok Byun 2015 [29]	Saliva	Whole unstimulated saliva	5–10	Condition: saliva was collected from 10 to 12 am, after a mechanical tooth and tongue cleansing, and a rinse with distilled water Preservation: - Temporary storage: - Long-term storage: -	TRIzol reagent (Invitrogen, Carlsbad, CA, USA), miRNeasy Mini Kit (QIAGEN, Valencia, CA, USA)	NanoDrop (Thermo Scientific, Wilmington, DE, USA) Microarray (Human miRNA Microarray 8 × 60K V19.0, Agilent Technologies, Santa Clara, CA, USA) TaqMan MicroRNA Assay (Applied Biosystems, Foster City, CA, USA) using qRT-PCR (7500 Real-Time PCR System, Applied Biosystems, Foster City, CA, USA)
3	F. Zahran 2015 [28]	Saliva	Whole unstimulated saliva	-	Condition: subjects refrained from eating, drinking, using chewing gum for at least one and a half hour prior sampling Preservation: - Temporary storage: - Long-term storage: -	miRNeasy kit (Qiagen, Valencia, CA, USA)	NanoDrop ND-1000 spectrophotometer (NanoDrop Technologies Inc., Wilmington, DE, USA), qRT-PCR (Applied Biosystems, 7500 Real-Time PCR System, Foster City, CA, USA)
4	Martin Lundegard 2015 [27]	Saliva	Stimulated saliva	2	Condition: - Preservation: Saliva Incubation Mix Temporary storage: -room temperature for 1 h Long-term storage: −80 °C	RNeasy Micro Kit (Qiagen, Hilden, Germany)	NanoDrop ND-1000 spectrophotometer (NanoDrop Technologies), qRT-PCR (IQ5 Multicolor Real-Time PCR Detection System, Bio-Rad Laboratories Inc., USA)
5	Kai-Feng Hung 2016 [30]	Saliva	Whole unstimulated saliva	-	Condition: after mouth rinsing Preservation: - Temporary storage: all saliva samples were processed immediately Long-term storage: −80 °C	mirVana PARIS kit (Ambion, Austin, TX, USA)	TaqMan MicroRNA (Applied Biosystems, Foster City, CA, USA) using qRT-PCR
6	Mehmet Bugrahan Duz 2016 [31]	Saliva	Whole saliva	-	Condition: in the morning, stop eating, drinking and oral hygiene 1 h before Preservation: - Temporary storage: all saliva samples were processed immediately Long-term storage: −80 °C	mirVana PARIS kit (Ambion, Darmstadt, Germany)	NanoDrop ND-2000c (Thermo Fisher Scientific Inc., Wilmington, DE, USA), Microarray (Agilent Technologies, Santa Clara, CA, USA), TaqMan MicroRNA (Applied Biosystems, Foster City, CA, USA) using qRT-PCR (LightCycler 480-II Real-Time PCR System, Roche, Switzerland)
7	Minoo Shahidi 2017 [32]	Saliva and tissue	Whole unstimulated saliva	5	Condition: refrain from consuming food, drinking and oral hygiene procedures for at least 1 h prior to sampling Preservation: RNA Stabilization Reagent Temporary storage: - Long-term storage: −80 °C	mirVana PARIS Kit (Ambion, USA)	Nanodrop ND-1000 (Thermo Scientific, Worcester, MA, USA), qRT-PCR (Rotor-Gene 6000 Real-Time PCR System, Corbett Life Science)
8	T.N. Uma Maheswari 2017 [33]	Saliva	Whole saliva	8–10	Condition: saliva was collected after a rinse with water, between 6 and 11 am, refrain from eating, drinking or applying oral hygiene in that day Preservation: - Temporary storage: dry ice Long-term storage: −80 °C	RNeasy Kit (Qiagen)	Nanodrop spectrophotometer, TaqMan MicroRNA (Applied Biosystems) using qRT-PCR
9	Kavan Shah 2018 [36]	Saliva and serum	Whole saliva	-	Condition: - Preservation: - Temporary storage: - Long-term storage: -	TRIzol reagent (Invitrogen, Carlsbad, CA, USA)	qRT-PCR (AriaMx Real-Time PCR System, Agilent Technologies)
10	Masoumeh Mehdipour 2018 [37]	Saliva	Whole unstimulated saliva	3–5	Condition: saliva was collected after a rinse with water; subjects refrained from eating, drinking or using oral hygiene procedures for at least 1h prior sampling; Preservation: RNAlater Temporary storage: - Long-term storage: −80 °C	mirVana PARIS kit (Ambion, USA)	qRT-PCR
11	Sana Maher Hasan Aghbari 2018 [34]	Saliva and tissue	Whole unstimulated saliva	-	Condition: - Preservation: - Temporary storage: - Long-term storage: −80 °C	miRNeasy extraction kit (Qiagen, Valencia, CA, USA)	qRT-PCR (Rotor-Gene Q Real-time PCR System, Qiagen, USA)
12	Chiara Gai 2018 [35]	Saliva	Whole unstimulated saliva	-	Condition: saliva was collected after a rinse with water, between 9 and 11 am, refrain from eating, drinking or applying oral hygiene Preservation: - Temporary storage: - Long-term storage: −80 °C	mirVana Isolation kit (Thermo Fisher Scientific, Waltham, MA, USA)	Nanodrop ND-1000 (Thermo Fisher Scientific), TaqMan MicroRNA (Thermo Fisher) using qRT-PCR (QuantStudio 12k Flex, Thermo Fisher)
13	Tami Yap 2018 [38]	Saliva and tissue	Oral swirl	10	Condition: patients were asked to swirl deionized water 50–60 s Preservation: - Temporary storage: Long-term storage: −20 °C	mirVana Isolation Kit kit (Life Technologies, USA)	NanoDrop 1000 (NanoDrop Technologies Wilmington, DE, USA), qRT-PCR (Eco Real-Time PCR System, Illumina)
14	Tami Yap 2019 [39]	Saliva	Oral swirl	10	Condition: patients were asked to swirl deionized water 50–60 s Preservation: - Temporary storage: - Long-term storage: -	mirVana Isolation Kit (Life Technologies)	NanoDrop 1000 (NanoDrop Technologies Wilmington, DE, USA), qRT-PCR (Eco Real-Time PCR System, Illumina)
15	Shesha R. Prasad 2020 [41]	Saliva	Whole unstimulated saliva	0.5–1.0	Condition: saliva was collected from 9 to 11 am, after a rinse with distilled water for 1 min; subjects were refrained from eating, drinking, smoking, or using oral hygiene procedures for at 1 h prior sampling; Preservation: RNAlater Temporary storage: - Long-term storage: −20 °C	miRNeasy kit (Qiagen)	qRT-PCR (Applied Biosystems StepOne Real-Time PCR System)
16	Uma Maheswari 2020 [40]	Saliva	Whole saliva	-	Condition: refrain from consuming food for 2 h prior to sampling, and avoid the use of chewing tobacco or smoking for 1 day before the collection of samples; Preservation: - Temporary storage: DNAse/RNAse-free 50 mL falcon containers at room temperature for maximum 3 h; Long-term storage: −4 °C	NucleoSpin^®^ miRNA (Macherey-Nagel, Germany)	qRT-PCR (Rotor-Gene Q 5-plex, Qiagen)
17	Zhichao Meng 2021 [47]	Saliva, tissue and serum	Whole saliva	-	Condition: - Preservation: - Temporary storage: - Long-term storage: −80 °C	TRIzol kit (Thermo Fisher)	qRT-PCR
18	Cheng Ann-Joy 2021 [42]	Saliva	Oral swabbing	-	Condition: oral swabbing was soaked in 1 mL of normal saline for over 30 min Preservation: - Temporary storage: - Long-term storage: -	miRNeasy Mini Kit (Qiagen, Valencia, CA, USA)	qRT-PCR (Bio-Rad CFX96 Real-Time PCR System, Bio-Rad, Hercules, CA, USA)
19	Chiara Romani 2021 [43]	Saliva	Whole unstimulated saliva	-	Condition: saliva was collected after a rinse with PBS, refrain from eating, drinking or applying oral hygiene Preservation: - Temporary storage: immediately processed Long-term storage: −80 °C	miRNeasy Mini Kit (Qiagen, Hilden, Germany)	Microarray (Agilent Technologies, Santa Clara, CA, USA) and qRT-PCR (Bio-Rad CFX96 Real-Time PCR System, Bio-Rad, Hercules, CA, USA)
20	Nikolay Mehterov 2021 [44]	Saliva Tissue	Whole unstimulated saliva	4–5 mL	Condition: saliva was collected after a rinse with distilled water, between 8 and 9 am, refrain from eating, drinking, smoking or applying oral hygiene Preservation: - Temporary storage: immediately pro-cessed Long-term storage: −80 °C	TRI Reagent (Invitrogen, ThermoFisher Scientific, Massachusetts, MA, USA)	NanoDrop 2000 (Thermo Scientific, Massachusetts, MA, USA) TaqMan MicroRNA (Thermo Fisher Scientific, Massachusetts, MA, USA) using qRT-PCR (Rotor-Gene Q Real-Time PCR System, Qiagen, Hilden, Germany)
21	Parma Kumari 2021 [45]	Saliva	Whole unstimulated saliva	-	Condition: fasting saliva via PureSAL device Preservation: - Temporary storage: - Long-term storage: −80 °C	mirVANA PARIS kit (Invitrogen, Thermo Fisher Scientific, USA)	TaqMan MicroRNA Assay Kit (Thermo Fisher Scientific, USA) using qRT-PCR (QuantStudio™ 7 Flex Real-Time PCR System, Applied Biosystems, USA)
22	Saba Khan 2021 [46]	Saliva	Whole saliva	-	Condition: - Preservation: - Temporary storage: - Long-term storage: −80 °C	-	qRT-PCR
23	Beáta Scholtz 2022 [50]	Saliva	Whole unstimulated saliva	5 mL	Condition: saliva was collected between 9 and 11 am, refrain from eating, drinking, smoking, gum chewing or applying oral hygiene for at least 60 min before sampling Preservation: PAXgene reagent Temporary storage: - Long-term storage: −70 °C	TRIzol Reagent (Invitrogen, Thermo Fisher Scientific, Waltham, MA, USA)	TaqMan Mi-croRNA (Applied Biosystems) using qRT-PCR (ABI PRISM^®^ 7000 Sequence Detection System, Applied Biosystems)
24	Dario Di Stasio 2022 [49]	Saliva	Whole unstimulated saliva	-	Condition: saliva was collected from 8 to 12 am Preservation: - Temporary storage: all saliva samples were processed within the shortest time Long-term storage: -	mirVana PARIS KIT (Thermo Fisher Scientific, Waltham, MA, USA)	TaqMan MicroRNA Assay (Life Technologies, Carlsbad, CA, USA) using qRT-PCR (ViiA™ 7 Real-Time PCR System, Applied Biosystems, Foster City, CA, USA)
25	Hsi-Feng Tu 2022 [48]	Saliva	Whole unstimulated saliva	2	Condition: saliva was collected from 9 to 10 am with prior mouth rinsing with water and the patients refrained from eating, drinking, smoking or using oral hygiene applying oral hygiene for at least 60 min before sampling Preservation: protease inhibitors Temporary storage: all saliva samples were processed within the shortest time Long-term storage: −80 °C	mirVana PARIS KIT (Ambion, Thermo Fisher Scientific, Austin, TX, USA)	TaqMan MicroRNA Assay (Applied Biosystems, Foster City, CA, USA), using qRT-PCR (ABI Prism 7700 Sequence Detection System, Applied Biosystems, Foster City, CA, USA)
26	Aditi Patel 2022 [51]	Saliva Tissue	Whole unstimulated saliva	-	Condition: saliva was collected between 8 and 13 pm, refrain from eating or applying oral hygiene for at least 60 min before sampling Preservation: - Temporary storage: - Long-term storage: −80 °C	TRIzol LS reagent (Thermo Fisher Scientific, Waltham, MA, USA)	TaqMan MicroRNA Assays (Applied Biosystems, Foster City, CA, USA), using RT-PCR (ABI Prism 7700 qPCR System, Applied Biosystems, Foster City, CA, USA)
27	Cosmin Ioan Faur 2022 [52]	Saliva	Whole unstimulated saliva	0.8–1.6	Condition: saliva was collected between 7 and 10 am, refrain from eating, drinking or applying oral hygiene for at least 60 min before sampling, after a rinse with water 15 min before sampling Preservation: - Temporary storage: −20 °C Long-term storage: −80 °C	Plasma/Serum Circulating and Exosomal RNA Purification Kit (Norgen Biotek Corp., Thorold, ON, Canada)	TaqMan Mi-croRNA (Invitrogen/Thermo Fisher Scientific, Waltham, MA, USA) using qRT-PCR (ViiA7 Real-Time PCR System, Applied Biosystems, Waltham, MA, USA)
28	Masoumeh Mehdipour 2023 [53]	Saliva	Whole unstimulated saliva	3–5	Condition: saliva was collected between 8 and 13 pm, refrain from eating, drinking or applying oral hygiene for at least 60 min before sampling Preservation: RNAlater Temporary storage: - Long-term storage: −80 °C	TRIzol Reagent (Anacell Teb, Tehran, Iran)	NanoDrop One (Thermo Scientific/Thermo Fisher, Waltham, MA, USA), qRT-PCR (Roche, Switzerland)
29	Aarushi Garg 2023 [54]	Saliva	Whole unstimulated saliva	3–5	Condition: - Preservation: RNAlater Temporary storage: - Long-term storage: −80 °C	QIAzol Lysis Reagent and miR-Neasy Mini Kit (Qiagen, Germany)	NanoPhotometer P330 (Implen / Biochrom International, Delhi, India), qRT-PCR (Rotor-Gene Q Real-Time PCR System, Qiagen, Germany)
30	Nayroz Abdel Fattah Tarrad 2023 [55]	Saliva	Whole unstimulated saliva	-	Condition: saliva was collected in the morning Preservation: - Temporary storage: −20 °C Long-term storage: -	-	qRT-PCR (Rotor-Gene Q real-time PCR system, Qiagen, USA)
31	Alieh Farshbaf 2024 [56]	Saliva	Whole saliva	-	Condition: saliva was collected in the morning, without eating or drinking Preservation: - Temporary storage: - Long-term storage: −80 °C	RNX-Plus (SinaClon, Tehran, Iran)	NanoDrop 2000 (Thermo Fisher Scientific, Wilmington, DE, SUA), qRT-PCR (LightCycler 96, Roche Diagnostics, Basel, Switzerland)
32	Federica Rocchetti 2024 [57]	Saliva Plasma	Whole saliva	2	Condition: saliva was collected and patients refrained from eating, drinking or consuming chewing gum for at least 90 min before sampling Preservation: RNA preservation Temporary storage: 16–24 °C Long-term storage: −80 °C	Saliva RNA purification kit (Norgen Biotek Corp., Thorold, ON, Canada)	TaqMan Mi-croRNA (Thermo Fisher Scientific, Waltham, MA, USA) using qRT-PCR (7500 Fast Real-Time PCR System, Thermo Fisher Scientific, Waltham, MA, USA)
33	Moataz M ELHefny 2024 [58]	Saliva	Whole unstimulated saliva	0.4	Condition: saliva was collected in the morning, refrain from eating, drinking or smoking for at least 30 min before sampling Preservation: - Temporary storage: on ice Long-term storage: −80 °C	MiRNeasy extraction kit (Qiagen Valencia, CA, USA)	qRT-PCR (Rotor-Gene Q PCR system, Qiagen Hilden/USA office, USA)

**Table 5:** The accuracy of using microRNAs as a method of distinction between OPMD and OSCC vs healthy individuals.

Author	OPMD Analyzed	Reference Probe	Salivary miRNA	Sensitivity	Specificity
Uma Maheswari [40]	Leukoplakia OLP OSMF OSMF + leukoplakia	Healthy controls	miR-21	66%	69%
		Healthy controls	miR-31	36%	40%
F. Momen-Heravi [26]	OLP	OSCC	miR-27b	85.71%	100%
Dario Di Stasio [49]	Dysplastic lesions	Healthy controls	miR-181b	94.1%	81.2%
F. Zahran [28]	OPMD with or without dysplasia	Healthy controls	miR-21	90%	60%
		Healthy controls	miR-184	75%	80%
		Healthy controls	miR-145	70%	60%
Sana Maher Hasan Aghbari [41]	OLP	Healthy controls	miR-27b	75%	100%
			miR-137	100%	80%
Zhichao Meng [47]	OLP	Healthy controls	miR-142-3p	75%	86.4%
Cheng Ann-Joy 2021 [42]	OPMD	Healthy controls	miR-196b	90.0%	98.1%
Chiara Romani 2021 [43]	OSCC	Healthy controls	miR-423-5p + miR-106b-5p, miR-193b-3p	85.4%	85.1%
Nikolay Mehterov 2021 [44]	OSCC	Healthy controls	miR-30c-5p	86%	74%
Hsi-Feng Tu 2022 [48]	OPMD	Healthy controls	miR-375	82%	80%
	OPMD in dysplasia state	OPMD in non-dysplasia state		71%	83%
Beáta Scholtz 2022 [50]	OSCC	Healthy controls	miR-31-5p, miR-345-3p, miR-424-3p	77%	86%
Aditi Patel 2022 [51]	OSCC	Healthy controls	miR-1307-5p	99.99%	99.99%
Cosmin Ioan Faur 2022 [52]	OSCC	Healthy controls	miR-486-5p	72%	59%
Aarushi Garg 2023 [54]	OSCC	Healthy controls	miR-21	80%	70%
	OPMD	Healthy controls		70%	53%
	OPMD	OSCC		70%	60%
	OSCC	Healthy controls	miR-184	80%	74%
	OPMD	Healthy controls		70%	60%
	OPMD	OSCC		70%	60%
Nayroz Abdel Fattah Tarrad 2023 [55]	OSCC	Healthy controls	miR-106a	100%	70.8%
	OLP	Healthy controls		83.5%	50%
	OLP	OSCC		66.7%	83.3%
Moataz M ELHefny 2024 [58]	OSCC	Healthy controls	miR-93	100%	100%
	OLP	Healthy controls		95%	95%
	OSCC	Healthy controls	miR-412-3p	100%	100%
	OLP	Healthy controls		100%	100%

## Data Availability

Not applicable.

## Supplementary Materials

The following supporting information can be downloaded at https://www.mdpi.com/article/10.3390/jcm14228128/s1, PRISMA checklist.

## Abbreviations

The following abbreviations are used in this manuscript:

OPMD	Oral potentially malignant diseases
QUADAS-2	Diagnostic Accuracy Research Tool
OSCC	Oral squamous cell carcinoma
OSMF	Oral submucous fibrosis
OLP	Oral lichen planus
OLL	Oral lichenoid lesion
OED	Oral epithelial dysplasia
Interleukin-6	Interleukin-6
CRP	C-reactive protein
TME	Tumor microenvironment
ECM	Extracellular matrix
CAF	Cancer-associated fibroblasts
GRα	Glucocorticoid receptor α
